# Feasibility of photon-counting spectral CT in dental applications—a comparative qualitative analysis

**DOI:** 10.1038/s41405-021-00060-x

**Published:** 2021-01-27

**Authors:** L. Vanden Broeke, M. Grillon, A. W. K. Yeung, W. Wu, R. Tanaka, V. Vardhanabhuti

**Affiliations:** 1 Department of Diagnostic Radiology, Li Ka Shing Faculty of Medicine, University of Hong Kong, Hong Kong SAR, China; 2 Oral and Maxillofacial Radiology, Applied Oral Sciences and Community Dental Care, Faculty of Dentistry, University of Hong Kong, Hong Kong SAR, China

**Keywords:** Dental radiology, Gutta percha, Dental pulp

## Abstract

**Purpose:**

The goal of this study was to demonstrate the feasibility of using photon-counting spectral CT for dental applications. This paper qualitatively analyzes the visibility of accessory canals (ACs) and metal artefacts from dental implants for cone-beam CT (CBCT), microtomography (microCT), and photon-counting spectral CT (PCSCT).

**Materials and methods:**

All of the teeth in this study were extracted, and eight teeth in total were scanned on a CBCT scanner, a microCT scanner and on a PCSCT scanner. Six of the teeth that were scanned have accessory canals, one has a titanium rod attached to it, and one has a gutta-percha point inside it. Qualitative analysis was done to compare the different imaging modalities.

**Results:**

The subjective image analysis demonstrated similar performance in AC detection and visualisation for PCSCT and CBCT (*p* value >0.05). Both PCSCT and microCT performed similarly for metal artefact reduction, and both were superior to CBCT (*p* value <0.05).

**Conclusion:**

Although microCT provides detailed information about small anatomical structures, it is not suitable for in vivo use. However, the PCSCT scanner was able to detect small anatomical structures in teeth comparable to CBCT, as well as being superior in reducing metal artefacts from dental implants. This study showed that PCSCT is a promising modality for future dentistry applications.

## Introduction

Computed tomography (CT) scanning has made a tremendous impact in medical applications and its uses have shown an exponential increase since the 1990s.^1^ In dental applications, the uses have mainly been in microCT, but technological innovation has remained relatively static. Recently, spectral scanning (using multiple energy bins) has been shown to be an important advancement in CT technology, particularly in material discrimination and artefact reduction.^2,3^ Advances in CT detectors, e.g. photon-counting detectors, which potentially improve spatial resolution compared to conventional CT detectors, are contributing to the emergence of spectral CT as a new imaging modality. With these new developments in mind, it is time to examine the use of novel spectral CT scanning in dentistry.

The MARS photon-counting spectral CT (PCSCT) scanner was designed and built by MARS Bioimaging Ltd. (MBI) (Christchurch, New Zealand). The MARS PCSCT scanner has energy discriminating capabilities and assigns incoming x-ray photons to one of eight, user-defined, energy bins (five energy bins are used for image reconstruction). Every x-ray photon is processed, therefore image quality is improved at a lower cost of radiation dose compared to conventional CT. A previous study has demonstrated that the MARS PCSCT scanner produces high-quality diagnostic images in humans without exceeding current clinical radiation dose levels (<5mGy), so it is suitable for in vivo human studies.^4^

Microtomography (microCT) systems have been widely used in multiple bioscience fields, as well as in dentistry. MicroCT is able to analyse various hard and soft tissue specimens with an excellent spatial resolution by generating voxels in the range of 5–50 μm.^5^ Therefore, microCT offers a noninvasive and precise analysis of root canal morphology. MicroCT, however, involves high radiation doses that are not compatible with in vivo human imaging.^6^ There are also technical limitations that limit the size of microCT systems. For these reasons, microCT is mostly used for ex vivo studies only.

Cone-beam CT (CBCT), one of the three-dimensional imaging modalities used in dentistry, has been reported to offer good accuracy with small voxel size, and reasonable costs and radiation dose compared to those known for conventional CT.^7–9^ Because of such favourable characteristics, CBCT has become the predominant imaging modality for various surgical treatments such as dental implantation.^9^ However, CBCT does not totally address the imaging needs in the oral and maxillofacial region. The reason for this is because orofacial structures are small, heterogeneous with different radiodensity, and in close proximity to each other. They also frequently have artificially inserted metallic objects which cause metal artefacts. Therefore, while CBCT exhibits excellent quality in outlining the bony structures in the orofacial region, its ability to visualise internal conditions of teeth and bones is not always optimal. The motivation of this paper is therefore to explore the use of PCSCT in dentistry applications as a proof-of-concept study.

The first part of this paper investigates root canal structures in teeth. The structure of root canals are complex and every detail of the root canal system needs to be considered in order to develop an appropriate plan for endodontic treatment. Sometimes, there are small structures spreading in various directions from the main canal. These structures are known as accessory canals (ACs). ACs can reach the outer surface of the root, establishing a direct relationship between the dental pulp and the periodontal space. The diameter of the ACs of primary molars has been found to range from 10 to 180 μm, with a median diameter of 67.0 μm.^10^ Therefore, ACs often go unnoticed.

With an optimised magnification and voxel size, the spatial resolution of the MARS PCSCT scanner is, theoretically, able to detect and visualise ACs. The first objective of this study is to qualitatively compare the ability of CBCT and PCSCT to detect and visualise ACs in teeth. MicroCT is considered the “radiological gold standard” and is therefore used as a reference. The second objective of this study is to qualitatively compare the presence of metal artefacts for CBCT, microCT and PCSCT. When x-rays pass through metal, effects such as beam hardening, photon starvation and partial volume cause artefacts.^11^ Metal artefacts are common in CBCT and they may interfere with the diagnostic process. PCSCT, on the other hand, has energy resolving capabilities and minimises metal artefacts in the reconstruction stage by dividing the spectrum into narrow energy bins.^3^ This paper focuses on the metal artefacts from two commonly used dental materials, one is a gutta percha point, and the other is a titanium implant. A gutta percha point is often used in root canal treatments. When a tooth suffers from an irreversible inflammation of the pulp, the clinician performs a root canal treatment during which the pulpal tissue is removed. The empty space is filled with a gutta percha point to prevent the ingression of bacteria. Because gutta percha points consist of metal sulfates, metal artefacts are often present when imaging the treated teeth using image modalities such as CBCT. This causes the gutta percha points to be inflated in diameter due to artefacts. With clear visualisation of the gutta percha point, without an inflated diameter, the clinician can better judge if the root filling procedure is of adequate quality or not. Titanium rods are a commonly used dental implant. They are surgically placed in the jawbone, where they serve as the roots of missing teeth. The evaluation of bone loss surrounding the dental implant is very useful. It is also useful to visualise the boundary of the implant and the jaw to see if the implant has slipped, or caused damage to the bone.

The purpose of this study is to investigate the use of the novel PCSCT scanner for dentistry applications. The performance of the MARS PCSCT scanner in (1) detecting and visualising ACs in teeth, and (2) reducing metal artefacts were qualitatively assessed and compared to CBCT and microCT. Although, the PCSCT scanner is not optimised for dentistry applications, this paper presents the first-ever explorative study of PCSCT for dentistry applications.

## Materials and methods

### Samples

Our samples consisted of six extracted teeth with known ACs. Five of these teeth have no metal fillings (teeth 1–5), and one tooth with known ACs has a metal filling (tooth 6). Tooth 1 was an incisor, tooth 2 a premolar and teeth 3, 4, 5 and 6 were molars. Teeth 7 and 8 were used for the assessment of metal artefacts, with one extracted tooth with a gutta percha point inside it, and one extracted tooth taped to a titanium rod. Each tooth was obtained from the Faculty of Dentistry, University of Hong Kong. The institutional ethics board has reviewed and approved the study. Eight teeth in total were scanned on a ProMax 3D Mid CBCT scanner (Planmeca Oy, Helsinki, Finland), a Skyscan 1172 microCT scanner (Skyscan, Aartselaar, Belgium), a Skyscan 1076 microCT scanner (Skyscan, Aartselaar, Belgium) and a MARS PCSCT scanner (MBI Ltd., Christchurch, New Zealand).

### Scanning technique

The scan parameters for the PCSCT scanner are shown in Table 1. The scan parameters for teeth 1–5, and for tooth 6 are different due to the latter having a metal filling. For tooth 6 and the dental implant studies, extrinsic filtration is added to the intrinsic 1.8 mm aluminium equivalent filtration to remove low energy photons. For the PCSCT images, only the highest energy bin images are displayed. The raw PCSCT images were processed with a Block-Matching and 3D filtering (BM3D) algorithm.^12^ However, only the highest energy bin images were used for analysis and displayed in this manuscript because these images were deemed the most diagnostically relevant.

**Table 1:** Scanning parameters used for the MARS PCSCT scanner.

Study	Tube voltage (kV)	Tube current (μA)	Exposure time (ms)	Filter	Energy thresholds (keV)	Voxel size (μm)
Teeth 1–5	80	53	300	None	20, 30, 45, 60	40
Tooth 6	120	23	160	2.5 mm aluminium	25, 32, 50, 79	40
Dental implants (teeth 7 and 8)	118	27	220	0.375 mm brass	45, 55, 65, 75	80

The energy thresholds are only shown for the charge summing mode (CSM) counters.

For the CBCT scans, each tooth was placed in the centre of a field-of-view of 4 × 5 cm (diameter × height), with exposure parameters of 90 kV and 12.5 mA, and an exposure time of 15 s. The voxel size was 75 μm. A noise reduction method is integrated into the CBCT scanner and uses the ARA algorithm from Planmeca to remove metal artefacts. For the microCT scans, each tooth (except for tooth 7) was placed in a cylindrical holder and scanned with Skyscan 1172 at 80 kV, 100 μA and with an exposure time of 4840 ms. MicroCT images of each tooth were acquired with an isotropic voxel size of 12.85 μm. Tooth 7 was scanned with Skyscan 1076 in the same cylindrical holder, with an exposure protocol of 88 kV and 100 μA and an exposure time of 560 ms. The voxel size was 17.33 μm. A “Despeckle” function on the CT scan software was applied to remove some artefacts.

### Qualitative image analysis

Qualitative image analysis was carried out for all of the studies by two board-certified dentists with 8 and 20 years’ experience. For teeth 1–6, subjective image quality was assessed for determining: (1) if the image is of diagnostic quality; (2) the visibility of the ACs; and (3) the image quality compared to microCT. Three orientations (axial, coronal and sagittal) for each of the six teeth (of approximately the same slice) were pre-selected by an experienced reader and presented to be compared and scored. MicroCT was considered the ground truth and was used as the reference standard (for the third scoring category). Readers were blinded to the other two imaging modalities (i.e. CBCT or PCSCT). The scoring system is outlined in Table 2. Note that some teeth have more than one AC, however, this analysis focuses on one AC only. The same AC is compared for the different imaging modalities.

**Table 2:** Scoring system for the determination of ACs.

Diagnostic quality		AC visualisation		Image quality	
1	Non-diagnostic	1	AC is definitely not present.	1	Severely inferior to microCT.
2	Equivocal	2	AC is unlikely to be present.	2	Substantially inferior to microCT.
3	Diagnostic	3	AC presence is equivocal.	3	Moderately inferior to microCT.
		4	AC is likely to be present.	4	Mildly inferior to microCT.
		5	AC is definitely present.	5	Similar to microCT.

For the metal artefacts study, two teeth with dental implants were scanned. One tooth had a cavity due to caries, and the other one had an exposed root canal system due to severe caries. The former was scanned with a titanium dental implant placed adjacent to it, whereas the latter had a gutta percha point inserted into its root canal. Images from the three modalities were also scored for determining the presence of metal artefacts for CBCT, microCT and PCSCT. In total, 18 images (axial, coronal and sagittal of approximately the same slice) were compared side-by-side with the scoring performed with the purpose of: (1) determining the diagnostic quality of the images; and (2) determining the visibility of metallic artefacts. Readers were blinded to the imaging modality type. The scoring system is outlined in Table 3. Note that the area of interest was the tooth-implant boundary.

**Table 3:** Scoring system for the reduction of metal artefacts.

Diagnostic quality		Artefacts	
1	Non-diagnostic	1	Massive obscuration of almost the entire area of interest.
2	Equivocal	2	Pronounced streaks obscuring a large area of interest.
3	Diagnostic	3	Minor streaks obscuring a small area of interest.
		4	Minimal streaks without significant obscuration of area of interest.
		5	Absence of artefacts.

The area of interest is the tooth-implant boundary. The assessments were performed by dentists with 8 and 20 years' experience.

### Viewing conditions

The teeth were viewed in a similar orientation for every modality. The selected images were then exported from their respective programs (OsiriX MD v.8.0.2, Planmeca Romexis 5.3.2.13 and the MARS Vision Software 2.0.9) in JPEG format, and displayed on a diagnostic grade monitor, Barco Coronis 2MP MDCG-2121 (Barco, Poperinge, Belgium). The readers were allowed to scroll the images, according to the criteria for assessment individually.

### Statistical analysis

The scores for the two readers were compared for statistical differences. For AC detection, the Wilcoxon matched-pairs signed rank test was performed to compare the performance between PCSCT and CBCT. For the dental implant study, a Kruskal–Wallis test was performed with post-test Dunn’s multiple comparisons. A *p* value of <0.05 implies statistical significance. All of the data were analysed using statistical software (Prism, version 8.4.3; GraphPad Software, San Diego, California, USA).

## Results

### AC detection

**Fig. 1:**
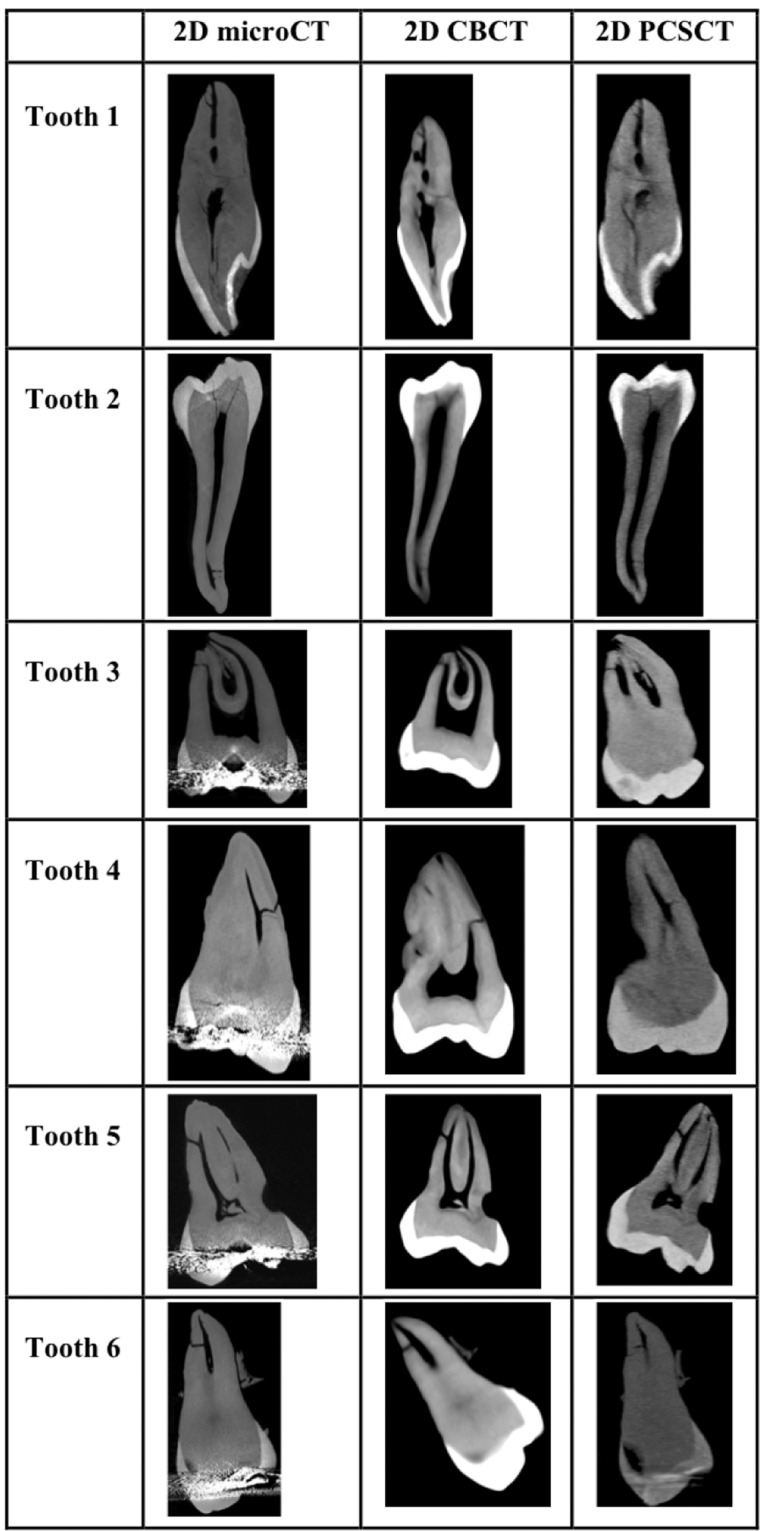
Representative 2D microCT, CBCT, and PCSCT images, for each tooth. The same AC is shown for each modality.

Six teeth with AC’s were scanned on a PSCT, a CBCT and on a microCT scanner. The microCT images were used as a reference for the scoring. Representative reconstructed microCT, CBCT and PCSCT sagittal images for each tooth are shown in Fig. 1. The scanning positions for each of the modalities was slightly different, hence each of the images has slightly different orientations.

The two readers scored the PCSCT and CBCT images following the scoring criteria in Table 2. Each reader has given 108 scores in total (three slice views, for three categories, for six teeth, for two modalities). A statistical analysis of the readers’ results is presented in Table 4. The mean, standard deviation and the standard error of the mean were calculated for the PCSCT images and the CBCT images. The mean scores for PCSCT and CBCT for diagnostic quality were 2.1 and 2.4, respectively. The mean scores for PCSCT and CBCT for AC visualisation were 3.5 and 3.7, respectively. The mean scores for PCSCT and CBCT for image quality were 2.9 and 3.1, respectively. The standard deviation and the standard error of the mean for PCSCT were slightly larger than for CBCT, for the three scoring categories.

**Table 4:** Results from the statistical analysis, for the three different scoring categories, for PCSCT and CBCT, for the AC detection part of the paper (teeth 1–6).

Scoring category	Mean (1 d.p.)		Standard deviation (1 d.p.)		Standard error of mean (1 d.p.)		Wilcoxon matched-pairs signed rank test *p* value
	PCSCT	CBCT	PCSCT	CBCT	PCSCT	CBCT	
Diagnostic quality	2.1	2.4	0.8	0.6	0.1	0.1	0.0931
AC visualisation	3.5	3.7	1.1	1	0.2	0.2	0.166
Image quality	2.9	3.1	1	1	0.2	0.2	0.2915

**Fig. 2:**
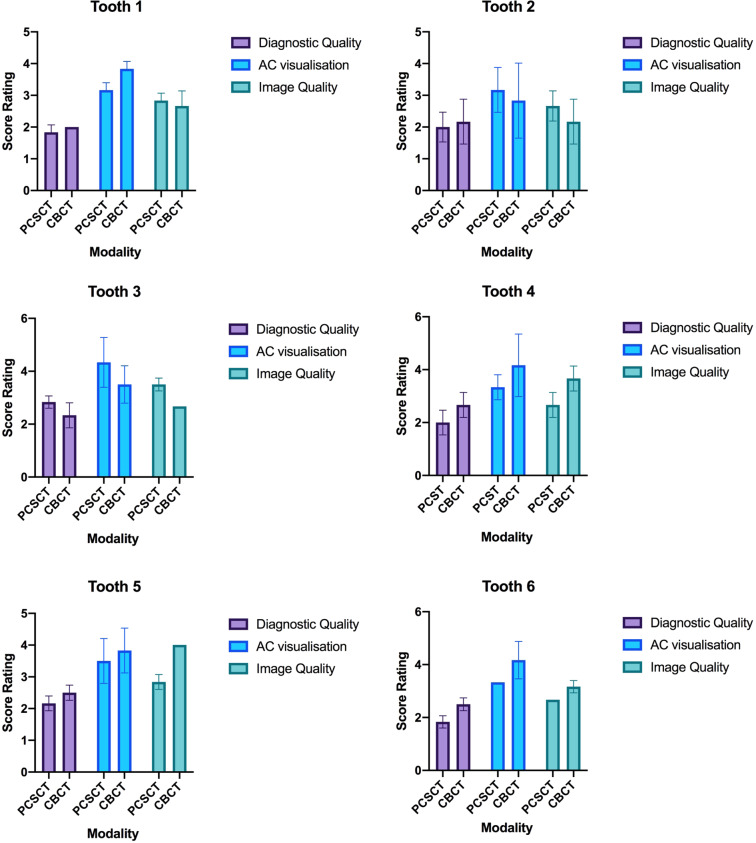
Graphical representation for AC visualisation in teeth 1–6. The error bars represent the difference in the scores of the two scorers.

**Fig. 3:**
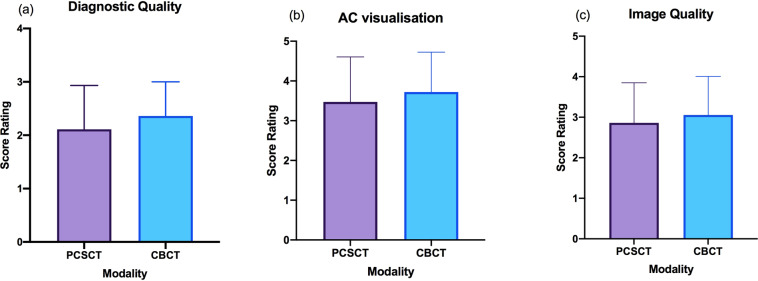
Qualitative assessment between PCSCT and CBCT. The average results from the two scorers for the six teeth for (**a**) diagnostic quality, (**b**) AC visualisation and (**c**) image quality.

A Wilcoxon matched-pairs signed rank test was performed to compare the performance between PCSCT and CBCT. There were no statistical differences between the two imaging modalities (*p* value >0.05). A graphical representation of the results are shown in Fig. 2 (averaged for all three slice views). Figure 3 shows a summary of the results in Fig. 2, averaged for teeth 1–6. Although the standard error of the mean for PCSCT is slightly bigger than for CBCT, this difference is not statistically significant (Table 4).

### Metal artefacts

**Fig. 4:**
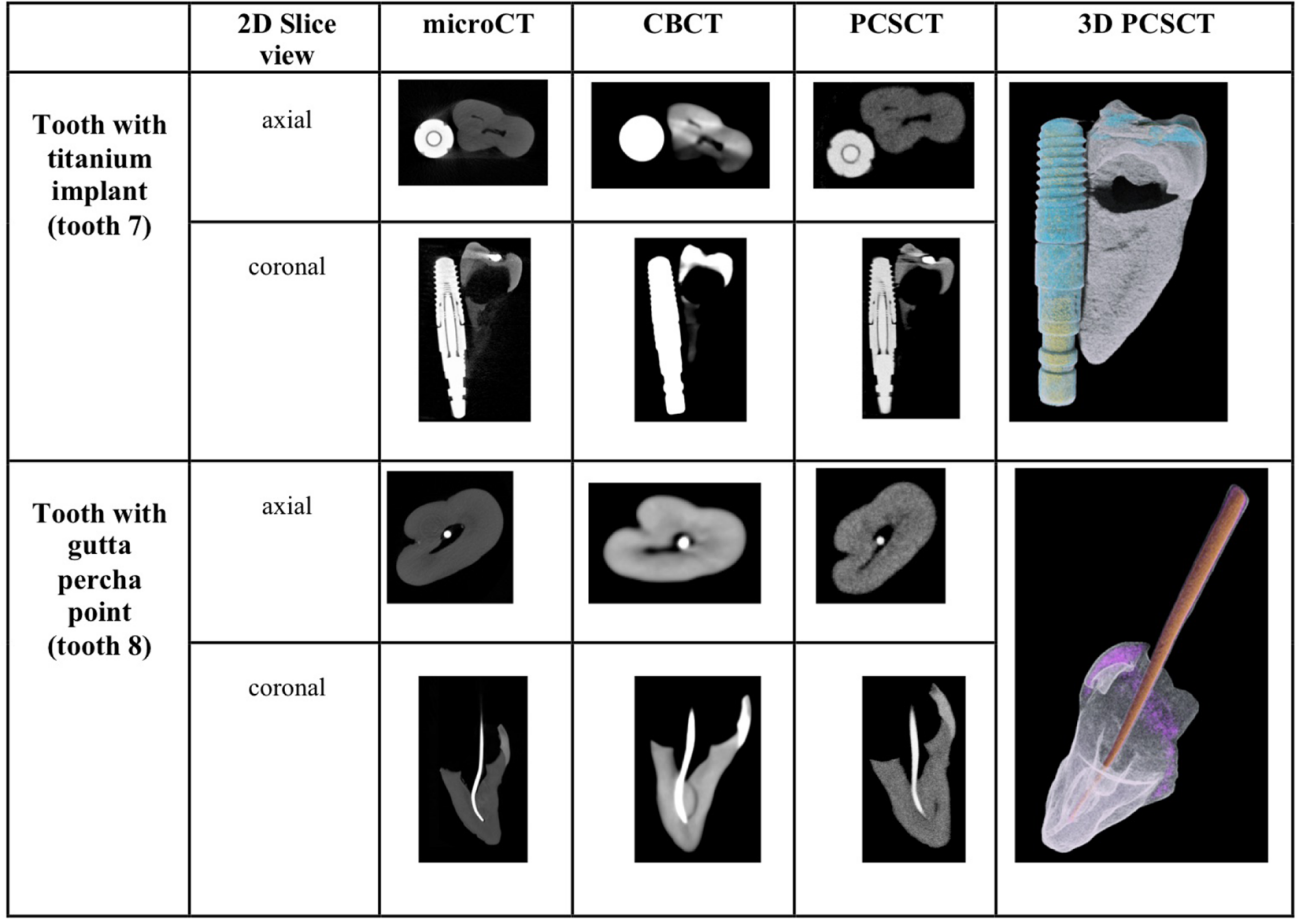
Representative 2D microCT, CBCT, and PCSCT image, for teeth 7 and 8. The 3D PCSCT images are also shown.

**Fig. 5:**
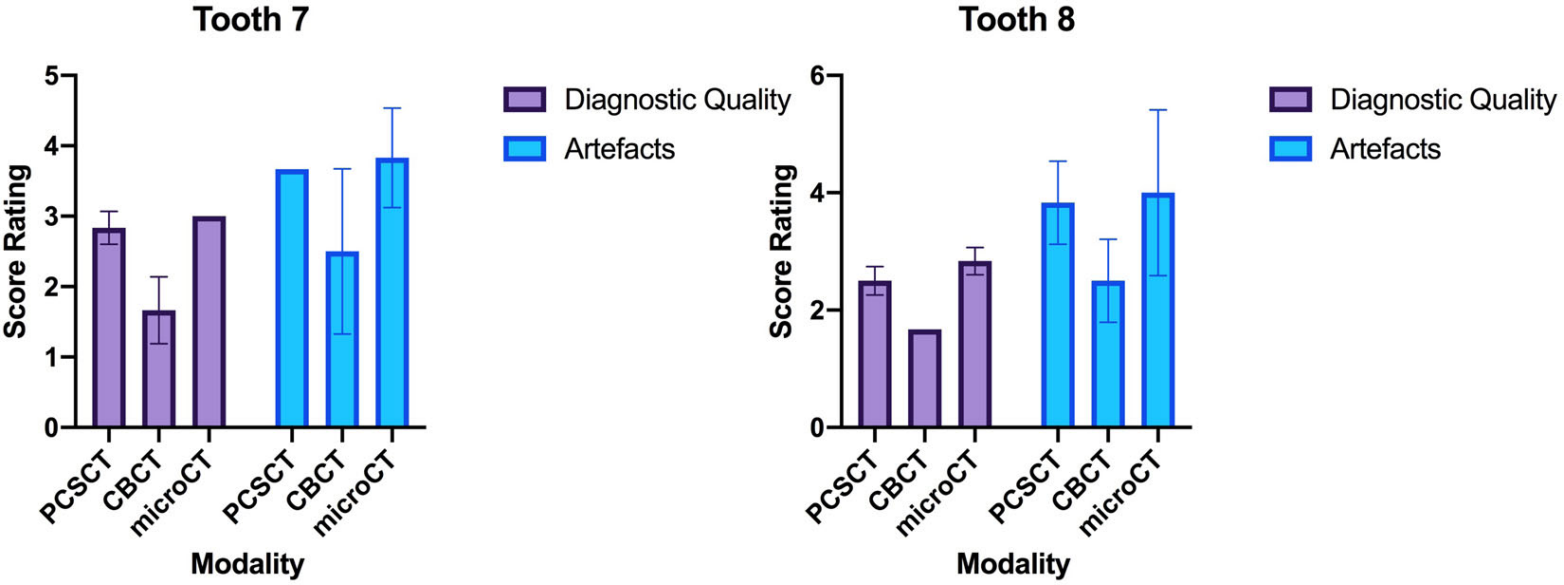
Qualitative analysis for tooth 7 (titanium rod) and tooth 8 (gutta percha point). The error bars represent the difference in the scores of the two scorers.

**Fig. 6:**
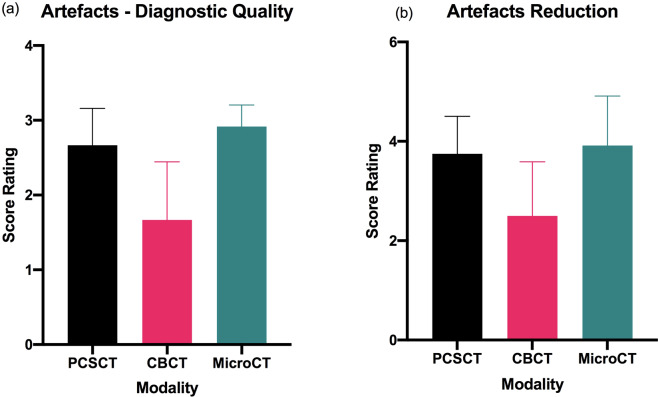
Summary results for artefacts assessment between PCSCT, CBCT and MicroCT. The average results from the two scorers for the two teeth, for (**a**) artefacts - diagnostic quality and (**b**) artefacts reduction.

Representative microCT, CBCT and PCSCT images for each tooth are shown in Fig. 4. The colour, three-dimensional volume-rendered PCSCT images are also shown. The two readers scored the microCT, PCSCT and CBCT images following the scoring categories in Table 3. Each reader has given 36 scores in total (three slice views, for two categories, for two teeth, for three modalities). The statistical analysis of the readers scores for the two teeth is shown in Table 5. For diagnostic quality, the mean scores for PCSCT, CBCT and microCT were 2.7, 1.7 and 2.9, respectively. For artefacts, the mean scores for PCSCT, CBCT and microCT were 3.8, 2.5 and 3.9, respectively. The results from the statistical analysis in Table 5 show that the two readers deemed the PCSCT images to be of similar quality to microCT (with similar mean scores), which were statistically superior to CBCT (*p* value <0.05). Table 5 shows that there was a statistically significant difference between PCSCT vs CBCT (*p* = 0.043), and CBCT vs microCT (*p* = 0.007), for the diagnostic category. For the artefacts category, there was a statistically significant difference between PCSCT vs CBCT (*p* = 0.013), and CBCT vs microCT (*p* = 0.009). The results from the statistical analysis are summarised in Fig. 5 and Table 5. Figure 6 shows a summary of the results between all three modalities.

**Table 5:** Results from the statistical analysis, for the two different scoring categories, for PCSCT, CBCT and microCT.

Scoring category	Mean (1 d.p.)			Standard deviation (1 d.p.)			Standard error of mean (1 d.p.)		
	PCSCT	CBCT	microCT	PCSCT	CBCT	microCT	PCSCT	CBCT	microCT
Diagnostic quality	2.7	1.7	2.9	0.5	0.8	0.3	0.1	0.2	0.1
Artefacts	3.8	2.5	3.9	0.8	1.1	1	0.2	0.3	0.3

All three imaging modalities are compared for the dental implant images (teeth 7 and 8).

## Discussion

The first part of this paper focused on the detection and the visualisation of ACs in teeth with CBCT and the novel MARS PCSCT scanner, using microCT as a reference. It is important for the clinician to be able to detect ACs as failure to do so may lead to a less-than-optimal endodontic treatment outcome.

In the first part of this study, we have shown that PCSCT and CBCT were equivalent in their ability to detect and visualise ACs. In the second part of this paper, we compared CBCT, PCSCT and microCT in metal artefact reduction from two types of dental implants (a titanium rod and a gutta percha point). We have shown that PCSCT compares well to the current reference standard of microCT, and was superior to CBCT.

MicroCT is the most diagnostically useful for AC detection and visualisation. However, microCT cannot be used in vivo due to technical limitation in system design as well as concerns regarding the large radiation doses. PCSCT, on the other hand, has a radiation dose equivalent to that of conventional CT. With already several dual-energy clinical scanners with or without photon-counting detectors in early clinical use in medical fields, it is anticipated that the PCSCT system will be widely available for human scanning in the near future. The MARS PCSCT scanner used in this study is a small animal scanner. However, a human scale MARS PCSCT scanner is based in Christchurch, New Zealand. The human scale scanner has the same capabilities as the small animal scanner used in this study. Dental applications of the MARS PCSCT human scale scanner will be investigated in the future. With this in mind, there are several potential in vivo applications which could be useful in dental radiology. First, as the CT data are isotropic with three-dimensional volumetric data, it would be possible, for example, to perform volumetric calculations of the root canal anatomy. Secondly, the evaluation of bone loss surrounding dental implant surfaces will be an important area for future exploration. The MARS PCSCT scanner is able to quantify how much of a specific material is in an area of interest, therefore it can be used to measure bone density. This feature was not utilised in this paper but would be useful for future studies. Thirdly, the alveolar bone surrounding a dental implant inserted into the jaws is often quite thin (in the range of several mm), and with the metal artefacts seen in conventional CT and CBCT, the bony information is often lost. With an accurate record of bone level surrounding the implant by imaging, the clinician can give a better judgement on the appropriate maintenance strategy and treatment options, and thus a better prognosis. CBCT has been the modality of choice for dentistry for two decades. It was designed to be used in dentistry and has been optimised for this purpose. PCSCT, on the other hand, is a new imaging modality and its many applications are still being discovered and refined. Once optimised, it is anticipated that better image quality can be achieved.

A few limitations of our study are worth noting. First, this is a proof-of-concept study, with a limited sample size. Second, for the PCSCT images, the visualisation and detection of the AC’s (teeth 1–6) were highly dependent on the scanning orientation. Once the scan was completed, the orientation of the slice views cannot be changed retrospectively. This is something that is currently being developed for future software releases of the MARS PCSCT visualisation system. On the contrary, for CBCT this feature was available. Third, we could not fully account to the readers’ inexperience of PCSCT as this is a new imaging modality. The readers were accustomed to analysing CBCT and microCT images. Fourth, we did not explore the effect of reconstruction or noise reduction algorithms specifically. Both CBCT and microCT have inbuilt algorithms to “smooth” the final images. Although the PCSCT images for the AC teeth (teeth 1–6) used the BM3D denoising algorithm (post-reconstruction), this process was not optimised and could be improved in the future. Finally, as the goal of this study was an early exploration in comparative assessment between PCSCT, CBT and microCT, radiation doses were not specifically measured, although the scanning parameters are comparable with conventional practices.

## Conclusion

The goal of this study was to demonstrate the feasibility of using PCSCT for dental applications. This paper was the first to perform a comparison between PCSCT, CBCT and microCT. Qualitatively, the ability of CBCT and PCSCT to visualise ACs performed similarly. For artefact reduction, microCT and PCSCT performed similarly with both being efficient at reducing the metal artefacts from the dental implants. Even though CBCT often has inbuilt metal artefact reduction algorithms, its performance is significantly inferior to microCT and PCSCT. This study has demonstrated some applications of PCSCT in dentistry and render PCSCT a potentially suitable modality for clinical use in dentistry in the near future.
